# The Contribution of Radial Endobronchial Ultrasound (EBUS) in the Diagnosis of Pulmonary Cystic Lesions

**DOI:** 10.7759/cureus.110073

**Published:** 2026-06-01

**Authors:** Élin Almeida, Gonçalo Samouco, Andreia Nunes, Luís Ferreia, Marcos Oliveira

**Affiliations:** 1 Pulmonology, Unidade Local de Saúde da Guarda, Guarda, PRT; 2 Faculty of Health Sciences, Universidade da Beira Interior, Covilhã, PRT

**Keywords:** bronchoscopy, cyst-associated lung cancer, multimodal diagnosis, peripheral pulmonary lesions, radial endobronchial ultrasound (r-ebus)

## Abstract

Neoplasms arising within pulmonary cystic airspaces are rare and diagnostically challenging entities that frequently lead to delayed recognition and advanced disease at diagnosis. We report the case of a 61-year-old smoker who presented with significant weight loss and two cystic pulmonary lesions on chest computed tomography (CT). An initial CT-guided transthoracic biopsy was non-diagnostic, and the patient subsequently underwent imaging surveillance. Follow-up CT demonstrated interval growth, progressive wall thickening, and spiculated margins, while ¹⁸F-fluorodeoxyglucose positron emission tomography/computed tomography (¹⁸F-FDG PET/CT) showed intense metabolic uptake, raising a strong suspicion for malignancy. Subsequent bronchoscopy with radial endobronchial ultrasound (r-EBUS) enabled real-time localization and transbronchial sampling of the lesion, confirming pulmonary adenocarcinoma. This case highlights the importance of maintaining a high index of suspicion for malignancy in cystic lung lesions, particularly in high-risk patients, and underscores the diagnostic value of r-EBUS as a safe, minimally invasive approach when conventional biopsy techniques are non-diagnostic.

## Introduction

Neoplasms associated with pulmonary cystic airspaces are rare, representing approximately 1%-4% of non-small cell lung cancers. They are characterized by a cystic or air-containing component with variable wall thickening, mural nodularity, or adjacent solid tissue. Their recognition remains challenging, and diagnostic delays are common, often resulting in presentation at more advanced stages [1]. Owing to their low prevalence and atypical imaging features, these lesions may initially be misinterpreted as benign conditions, including emphysematous bullae, inflammatory changes, or infectious processes [2]. Obtaining tissue can also be difficult because the diagnostically relevant component is often small, eccentric, or located adjacent to aerated cystic spaces, increasing the risk of non-diagnostic or non-representative sampling. In lung cancer screening populations, cystic lung lesions have been reported in around 1.5% of screened individuals, with malignancy ultimately diagnosed in around 14% of these cases [2]. Adenocarcinoma is the most frequent histological subtype, accounting for up to 88% of reported cases [3]. These features highlight the importance of maintaining a high index of suspicion and adopting a multimodal diagnostic approach. The inclusion of atypical pulmonary cysts in Lung-RADS v2022 (Lung Imaging Reporting and Data System, Version 2022) provides a structured framework for reporting and risk stratification, reflecting the growing recognition of these lesions in screening settings [4]. However, once malignancy is suspected, tissue confirmation remains essential and may be technically challenging. In this context, radial endobronchial ultrasound may provide additional value by improving lesion localization and guiding bronchoscopic sampling of the solid or thickened component associated with cystic pulmonary lesions.

## Case presentation

**Figure 1:**
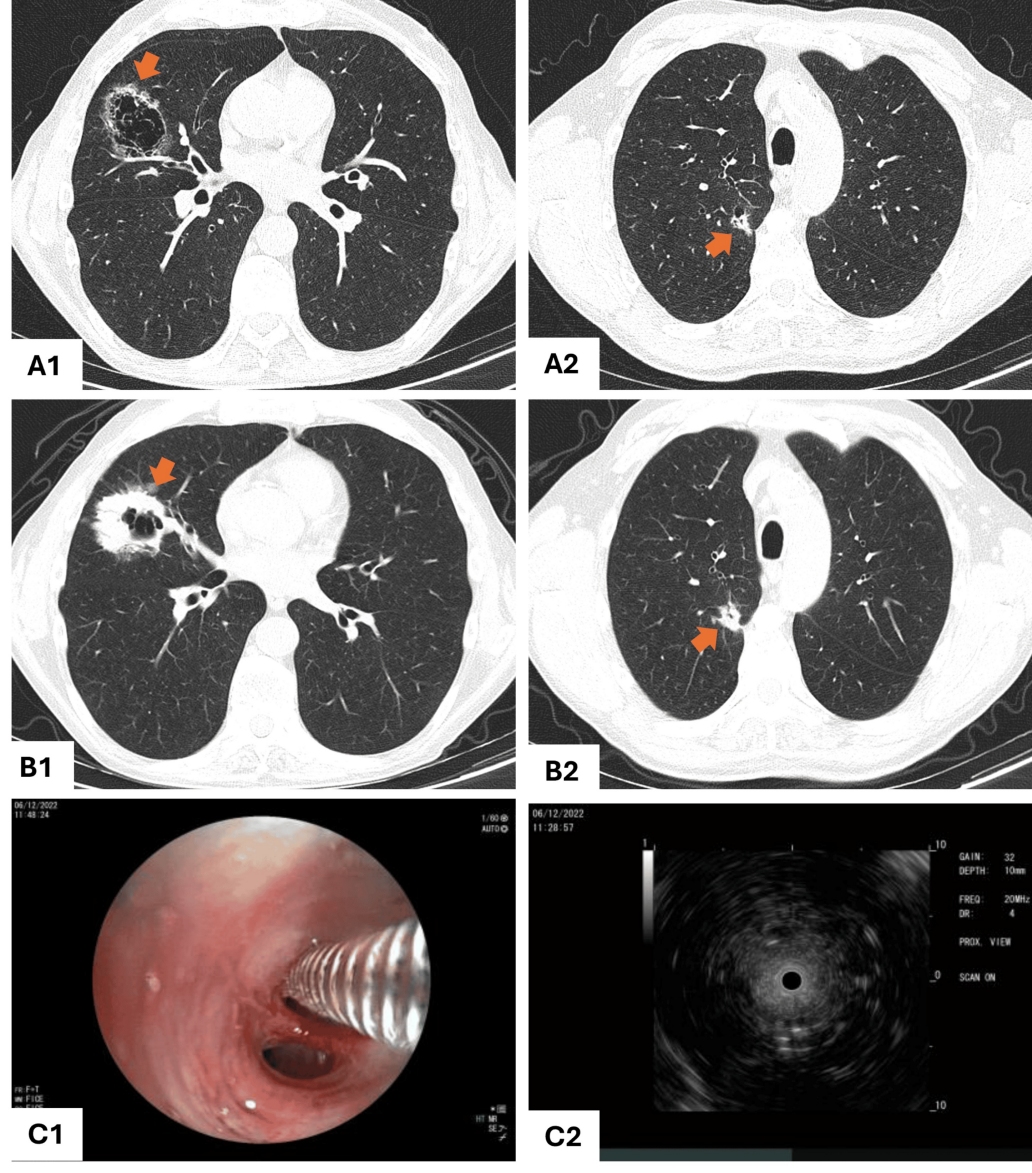
Serial chest CT and bronchoscopic assessment of progressive cystic lung lesions (A1, A2) Baseline axial chest CT showing a dominant cystic lesion in the middle lobe and a smaller cystic lesion in the right upper lobe. At baseline, the dominant lesion measured 42 mm, with a wall thickness of 5 mm and the presence of mural nodularity. (B1, B2) Follow-up CT after 12 months, showing interval enlargement with the dominant lesion measuring 56 mm, increased wall thickness, and development of spiculated margins. (C1) Bronchoscopic view of the segmental bronchus leading to the middle lobe lesion. (C2) Radial EBUS image demonstrating a concentric probe-within-lesion pattern. The miniprobe is centrally located within a circumferential solid lesion, which shows predominantly hypoechoic, heterogeneous echotexture with scattered internal hyperechoic foci and loss of the normal aerated lung artifact. EBUS: Endobronchial ultrasound.

We report the case of a 61-year-old man with a cumulative smoking history of 60 pack-years who presented with significant unintentional weight loss. Chest computed tomography (CT) revealed two cystic pulmonary lesions: a 42 mm thin-walled cystic lesion in the middle lobe and a 10 mm cystic lesion in the right upper lobe (Figure 1, Panels A1 and A2). Initial workup, including pulmonary function testing and sputum mycobacterial cultures, yielded unremarkable results. At baseline, the differential diagnosis included benign cystic airspace, emphysematous bulla, post-inflammatory cystic change, infectious cavity, and malignancy arising in a cystic airspace. The absence of microbiological evidence of infection, together with the patient’s smoking history and constitutional symptoms, supported the need for tissue diagnosis. A CT-guided transthoracic needle biopsy of the larger middle lobe lesion was performed but proved non-diagnostic. Surgical biopsy was subsequently recommended; however, the patient declined the procedure, and a strategy of close imaging surveillance was adopted.

At 12-month follow-up, repeat chest CT demonstrated enlargement of both lesions (from 42 to 56 mm and from 12 to 17 mm), with notable increases in wall thickness from 5 to 14 mm, and the development of new spiculated margins (Figure 1, Panels B1 and B2). ¹⁸F-fluorodeoxyglucose positron emission tomography/computed tomography (¹⁸F-FDG PET/CT) revealed intense metabolic uptake in both cystic lesions, with a maximum standardized uptake value (SUVmax) of 6.9 in the middle lobe lesion and 13.5 in the right upper lobe lesion, raising a strong suspicion for malignancy. After PET-positive progression, repeat CT-guided biopsy and surgical biopsy were considered. However, given the previous non-diagnostic percutaneous biopsy, the patient’s previous refusal of surgery, and the presence of bronchoscopic accessibility to the dominant lesion, r-EBUS-guided transbronchial biopsy was selected as a minimally invasive diagnostic alternative. During the procedure, r-EBUS localized the lesion and showed a concentric probe-within-lesion pattern, with the miniprobe centrally positioned within a circumferential solid lesion of predominantly hypoechoic and heterogeneous echotexture (Figure 1, Panels C1 and C2). r-EBUS-guided transbronchial biopsies were subsequently performed, yielding eight tissue samples. Rapid on-site evaluation was not available. Histopathological analysis of the biopsy specimens established the diagnosis of pulmonary adenocarcinoma. Table 1 presents a chronological summary of the diagnostic workup.

**Table 1:** Chronological timeline summarizing the key diagnostic steps PET/CT: Positron emission tomography/computed tomography; SUVmax: Maximum standardized uptake value.

Time Point	Findings/Intervention	Clinical Interpretation
Baseline	CT showed a 42-mm cystic lesion in the middle lobe and a 10-mm cystic lesion in the right upper lobe	Suspicious cystic lung lesions in a high-risk smoker
Baseline biopsy	CT-guided transthoracic biopsy of the dominant lesion was non-diagnostic	Malignancy not excluded
After biopsy	Surgical biopsy recommended; patient declined	Imaging surveillance adopted after shared decision-making
12-month follow-up	CT showed lesion growth, wall thickening, and spiculated margins	Radiological progression concerning malignancy
PET/CT	Intense FDG uptake in both cystic lesions, SUVmax 6.9 in the middle lobe and 13.5 in the right upper lobe	High suspicion for malignancy
Bronchoscopy	Radial EBUS localized the lesion; transbronchial biopsies were obtained	Targeted minimally invasive tissue sampling
Final diagnosis	Histology confirmed pulmonary adenocarcinoma	Diagnosis established

**​​​​​​​**After histological confirmation of pulmonary adenocarcinoma, the patient was referred for staging and multidisciplinary discussion. The final clinical stage was IIIA (T4N0M0), and neoadjuvant chemotherapy with carboplatin and pemetrexed was proposed. Following systemic treatment, the patient underwent right upper and middle lobectomy. Adjuvant therapy was not pursued because of poor performance status following a postoperative stroke. The patient subsequently experienced progressive clinical decline and died 18 months after diagnosis.

## Discussion

This case illustrates several important clinical considerations. First, cyst-associated pulmonary malignancies demand a high index of suspicion, particularly in patients with a significant smoking history and constitutional symptoms. The key radiological features that should prompt concern for malignant transformation are well supported by literature and include interval growth, progressive wall thickening, and the appearance of irregular or spiculated margins [5,6]. These warning signs must be systematically sought during surveillance imaging to minimize diagnostic delay. Second, this case highlights the valuable role of r-EBUS as a diagnostic tool for atypical peripheral pulmonary lesions. While CT-guided transthoracic biopsy remains the standard approach for peripheral lung lesions, with a higher pooled diagnostic yield (83%-93% vs. 69%-75% for r-EBUS), it carries a well-recognized risk of complications, most notably pneumothorax and hemorrhage [7,8]. In our patient, CT-guided biopsy failed to yield a definitive diagnosis. Radial EBUS offers a complementary minimally invasive approach that, although generally associated with a lower overall diagnostic sensitivity compared with CT-guided biopsy, provides a favorable safety profile with significantly fewer procedural complications [7]. Several factors can enhance r-EBUS diagnostic yield: lesion size > 2 cm, probe positioning within (rather than adjacent to) the lesion, the presence of a bronchus sign on CT, and the use of rapid on-site evaluation [9,10]. Its ability to localize peripheral lesions in real time enhances the precision of transbronchial sampling, making it particularly useful when initial percutaneous approaches are inconclusive or when patients are poor candidates for more invasive procedures.

## Conclusions

This case highlights the diagnostic complexity of pulmonary malignancies arising within cystic airspaces, particularly when early imaging features overlap with benign cystic or inflammatory lesions. It emphasizes the importance of careful longitudinal follow-up, with attention to specific morphologic changes predictive of malignant transformation such as interval growth, progressive wall thickening, mural nodularity, or spiculated margins. When histological confirmation is needed, a multimodal diagnostic strategy is essential. In selected patients with progressive cystic lung lesions, particularly when percutaneous biopsy is non-diagnostic or surgical biopsy is not feasible or is declined, r-EBUS-guided sampling may provide a useful minimally invasive diagnostic alternative. However, conclusions regarding comparative diagnostic yield cannot be drawn from a single case.
